# Optical and Electrical Properties of AlGaN-Based High Electron Mobility Transistors and Photodetectors with AlGaN/AlN/GaN Channel-Stacking Structure

**DOI:** 10.1021/acsomega.4c03082

**Published:** 2024-05-28

**Authors:** Chia-Feng Lin, Kun-Pin Huang, Han-Wei Wang, Kuei-Ting Chen, Cheng-Jie Wang, Yu-Cheng Kao, Hsiang Chen, Yung-Sen Lin

**Affiliations:** † Department of Materials Science and Engineering, National Chung Hsing University, Taichung 402202, Taiwan; ‡ Department of Applied Materials and Optoelectronic Engineering, National Chi Nan University, Nantou 54561, Taiwan; § Department of Chemical Engineering, Feng Chia University, Seatwen, Taichung 407, Taiwan

## Abstract

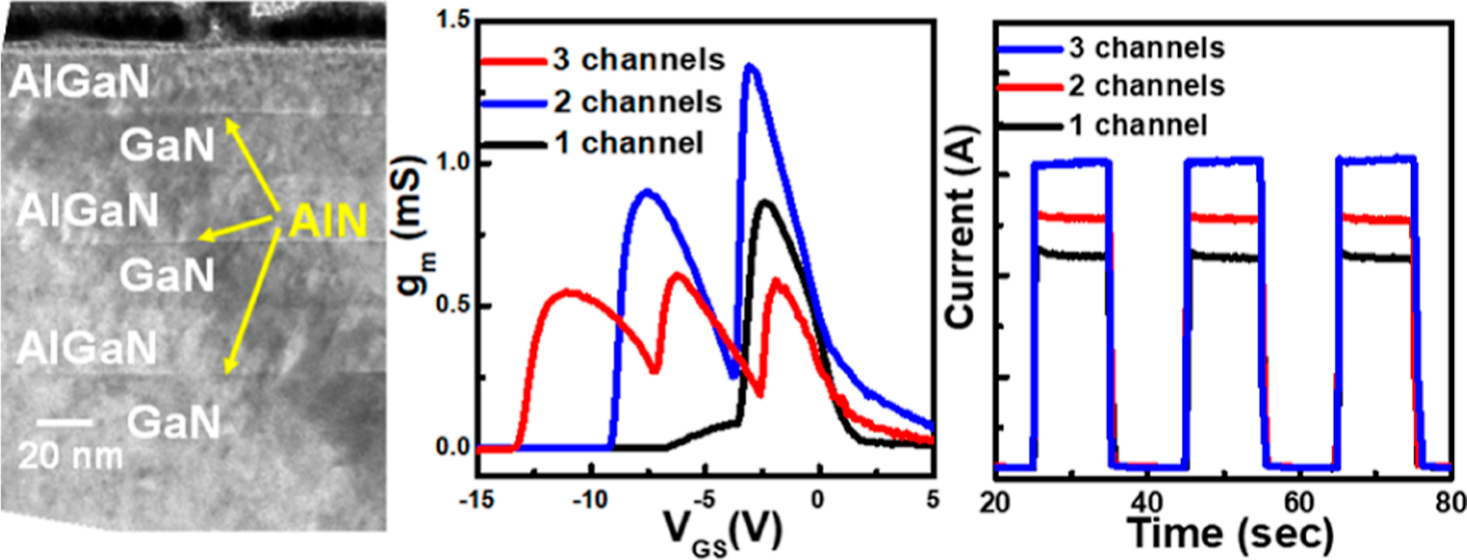

High channel current of the high electron mobility transistors (HEMTs) and high relative responsivity of the photodetectors (PDs) were demonstrated in the AlGaN/AlN/GaN channel-stacking epitaxial structures. The interference properties of the X-ray curves indicated high-quality interfaces of the conductive channels. The AlGaN/AlN/GaN interfaces were observed clearly in the transmission electron microscope micrograph. The saturation *I*_ds_ currents of the HEMT structures were increased by adding a number of channels. The conductive properties of the channel-stacking structures corresponded to the peaks of the transconductance (*g*_m_) spectra in the HEMT structures. The depletion-mode one- and two-channel HEMT structures can be operated at the cutoff region by increasing the reverse *V*_gs_ bias voltages. Higher *I*_ds_ current in the active state and lower current in the cutoff state were observed in the two-channel HEMT structure compared with one- and three-channel HEMT structures. For the channel-stacking metal–semiconductor–metal photodetector structures, the peak responsivity was observed at almost 300 nm incident monochromic light, which was increased by adding a number of channel layers. The channel current of the HEMT devices and the photocurrent in the PD devices were increased by adding a number of two-dimensional electron gas (2DEG) channels. By using a flat gate metal layer, the two-channel AlGaN/AlN/GaN HEMT structures exhibited a high *I*_ds_ current, a low cutoff current, and a high peak *g*_m_ value and have the potential for GaN-based power devices, fast portable chargers, and ultraviolet PD applications.

## 1 Introduction

Aluminum gallium nitride (AlGaN)-based materials have been extensively used as ultraviolet light-emitting diodes, photodetectors (PD),^1,2^ and high electron mobility transistors (HEMTs). AlGaN/GaN two-dimensional electron gas (2DEG) structures grown on the SiC substrate had been reported in our previous works.^3,4^ AlGaN/GaN double-channel structures to improve the transport properties in the HEMT devices had been grown on Al_2_O_3_,^5,6^ Si, GaN,^7^ and SiC^8,9^ substrates. A high UV-to-visible rejection ratio of AlGaN-based PDs^10,11^ has been reported. The electroluminescence and PD properties were shown in the Schottky-type p-GaN gate double-channel GaN HEMT structures.^12^ The p-GaN/AlGaN/GaN structure with a p-GaN gate and 2DEG channel had been reported for the visible-blind UV PDs.^13^ The self-powered photoelectrochemical-type PDs were reported using the Pt/AlGaN structure.^14^ The UV PD with a 2DEG channel at the AlGaN/GaN interface was fabricated as an interdigitated transducer.^15^ Multichannel AlGaN-based HEMTs had been demonstrated and well-controlled by using trigate structures,^16−18^ in which the effects of conductive channel regions were reduced. The HEMT and PD devices with flat metal electrodes were fabricated on the AlGaN/AlN/GaN channel-stacking structures to improve the channel current and photocurrent in this study.

In this paper, an AlGaN-based HEMT with AlGaN/AlN/GaN channel-stacking structures has been fabricated. High-quality AlGaN/AlN/GaN channel-stacking structures were observed in the transmission electron microscopy (TEM) image and the interference properties in the X-ray curves. The saturated *I*_ds_ currents were increased by adding a number of channel structures. The two-channel HEMT structure had a high *I*_ds_ current and a low pinch-off current under reverse gate bias voltage conditions. The switch-off properties of the AlGaN/AlN/GaN channel-stacking structures were observed clearly in the transconductance (*g*_m_) spectra. The optical, crystalline, and electrical properties of the channel-stacking HEMT structures were analyzed in detail.

## 2 Experimental Details

**Figure 1:**
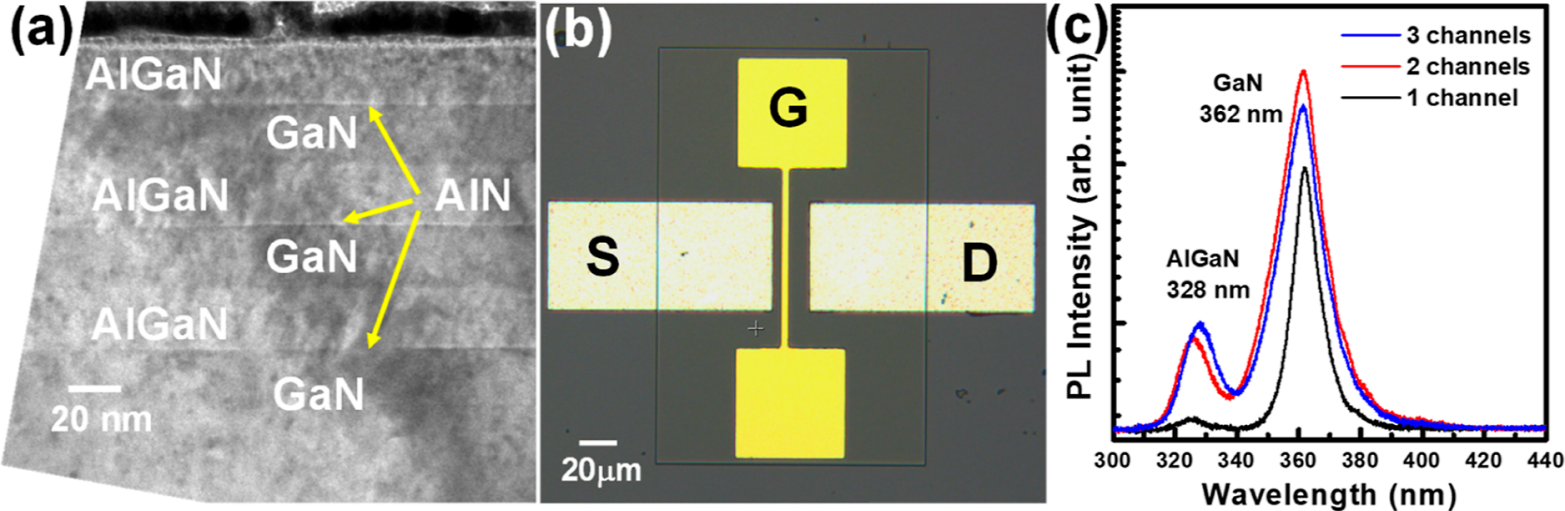
(a) TEM micrograph of the 3 pair AlGaN/AlN/GaN channel structure. (b) Optical microscopy image of AlGaN/AlN/GaN HEMT device. (c) PL spectra of the HEMT structures with different channel numbers.

The AlGaN/AlN/GaN channel-stacking HEMT structures were grown on flat sapphire substrates by using a metal–organic chemical vapor deposition (MOCVD) reactor. Trimethylgallium (TMGa), trimethylalane (TMAl), and ammonia (NH_3_) were used as Ga, Al, and N sources in the MOCVD system, respectively. The epitaxial structure consisted of an AlN buffer layer, an unintentionally doped GaN layer, a C-doped GaN insulated layer, an undoped-GaN/AlN/AlGaN (110/1/21 nm) channel structure, and a 5 nm thick u-GaN cap layer. For the channel-stacking structures, the two- and three-pair undoped GaN/AlN/AlGaN (22/1/21 nm) stack structures were inserted into the undoped GaN channel layer. The number of the 2DEG channels was added, and the total thickness of the active layer was fixed at 137 nm above the GaN/C insulated layer. The HEMT structure with three pairs of GaN/AlN/AlGaN stack structure is shown in Figure 1a. For the HEMT device fabrication, the mesa regions were defined through the photolithography process and Cl_2_-based inductively coupled plasma-reaction ion etching process. The Ti/Al ohmic contact layers were deposited across the mesa sidewall to form the source (S) and drain (D) metal pads. Then, the Ti/Al metal pads were thermally treated in the rapid thermal annealing process at 600 °C for 30 s to form ohmic contacts. The Ni/Au metal layers were deposited as the gate (G) electrode on the mesa region. The gate length, gate width, and S/D spacing of the HEMT structure were measured to be 3, 60, and 20 μm, respectively, as shown in Figure 1b. The metal–semiconductor–metal photodetectors (MSM-PD) were fabricated on the AlGaN/AlN/GaN channel-stacking epitaxial structure. The Ni/Au (10 nm/100 nm) Schottky metal contact bilayers were deposited on the epitaxial layers through the E-beam evaporation process and the photoresist lift-off process. The metal electrode patterns of the MSM structure are 200 μm in length, 9 μm in width, and 9 μm in spacing width. The size of the MSM detector is 800 μm × 280 μm.

An optical profilometer (Zeta-20) and a transmission electron microscope (TEM, JEM-2010, JEOL) were used to observe surface topography and microscopic images. The fabricated devices were analyzed by a precision semiconductor parameter analyzer (4156C). Photoluminescence (PL) spectra were analyzed through an imaging spectrometer (iHR550, HORIBA) and resolved using a 300 lines/mm grating, a thermoelectrically cooled CCD detector, and a 266 nm laser as the excited laser source. The crystalline properties were measured by X-ray diffraction (Bruker D8). The photoresponse and responsivity measurements of the AlGaN/AlN/GaN channel-stacking photodiodes were analyzed by using a Keithley 236 source meter and monochromatic illumination that was obtained by using a 500 W Xe lamp and a monochromator (JOBIN YVON iHR190) with a 5 nm spectral resolution.

## 3 Results and Discussion

Figure 1a shows the schematic and TEM microscopy images of a channel-stacking HEMT structure. The three pairs of undoped-GaN/AlN/AlGaN (22/1/20 nm) channel structures were observed in the TEM micrograph with Pt metal as the protected layer during the focused ion beam process for TEM sample preparation. The 2DEG channels were designed at the GaN layers close to the AlN/AlGaN bilayer structure in all HEMT structures. The TEM micrograph of the three-channel HEMT structure is shown in Figure 1a in which the detailed epitaxial structure for the AlGaN/AlN/GaN stack structure was observed. The 1 nm thick AlN is not easy to observe and the bright interfaces between the AlGaN and GaN layers were shown in the TEM micrograph. The large band inversion structure occurs at the interface between AlGaN/AlN bilayers and GaN channel layers, where the inversion band is lower than the Fermi level to form the 2DEG channel layers. The OM image of the HEMT device with a flat gate structure is shown in Figure 1b. The asymmetric structures of L_gs_ (5 μm) and L_gd_ (12 μm) were designed in the HEMT devices, in which the channels of the HEMT structures can be effectively controlled at the cutoff states. The conventional AlGaN-based HEMT structure had a 110 nm thick undoped GaN layer as the channel structure under the AlN/AlGaN epitaxial layers. For the three-channel HEMT structure, three pairs of the GaN/AlN/AlGaN stacking heterostructures were designed to replace the conventional channel structure. The PL spectra of all samples were measured as shown in Figure 1c. The PL peak wavelengths were measured at 362 nm for the GaN layer and 328 nm for the AlGaN layer in the channel region. The Al content of the AlGaN layer was calculated through the parabolic compositional dependence band gap of the alloys: *E*_g_,_AlGaN_ = *xE*_g_,_GaN_ + (1 – *x*)*E*_g_,_AlN_ – *bx*(1 – *x*); *E*_g_,_GaN_ = 3.413 eV, *E*_g_,_AlN_ = 6.2 eV, and *b* = 1.5.^19,20^ The Al content of the AlGaN layer was calculated as 0.24. The Al content of the AlGaN channel layer was calculated as 0.24 in the GaN/AlN/Al_0.24_GaN stack structures. By an increase in the number of the 2DEG channels, the PL intensities of the AlGaN layers were increased by inserting AlGaN stacking layers in the channel epitaxial structures. The PL peak of the AlGaN peaks had a slight red shift when the number of 2DEG channels was added. This could be explained by the strain-induced Al content slightly reducing in the AlGaN layer in the stack-channel structure during the epitaxial growth process.

**Figure 2:**
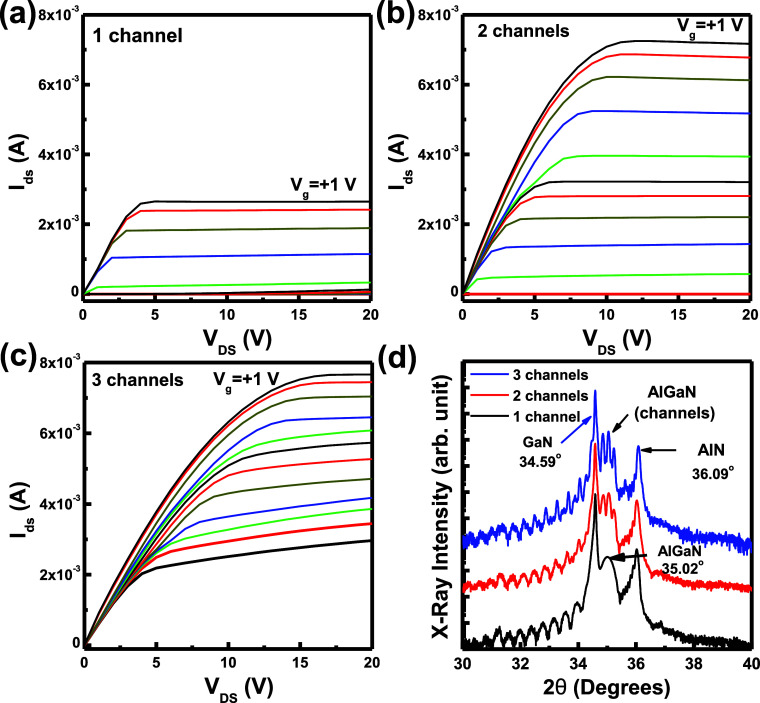
*I*_ds_–*V*_ds_ curves of the (a) one-channel, (b) two-channel, and (c) three-channel HEMT structures were measured by varying the gate bias voltages from +1 to −10 V. (d) X-ray rocking curves of the HEMT structures with different channel numbers were measured.

In Figure 2, the *I*_ds_ current as a function of the *V*_ds_ voltage was measured for the HEMT devices by varying the gate voltage (*V*_gs_) from +1 to −10 V. At 20 V of *V*_ds_ voltage, the *I*_ds_ currents were measured as 2.65 mA for one-channel HEMT, 7.17 mA for two-channels HEMT, and 7.66 mA for three-channels HEMT structures at +1 V gate voltage as shown in Figure 2a–c. The maximum *I*_ds_ of the HEMT devices was operated in the active region, in which the operating conditions were +20 V for *V*_ds_ and +1 V for *V*_gs_. The maximum *I*_ds_ currents were increased by adding a number of 2DEG channels in the AlGaN/AlN/GaN stack structures. The large *I*_ds_ current of the three-channel structure was observed at the active region at *V*_ds_ = +20 V. In the three-channel HEMT structure, the leakage current was observed at +20 V for *V*_ds_ and reversed *V*_gs_ bias conditions. The three-channel HEMT structure cannot operate at the cutoff region, which was observed in Figure 2c. The conductive channels of both one-channel and two-channel HEMT structures can be closed and pinched-off at the cutoff region. To improve the electrical properties of the HEMT devices, the thickness of the AlGaN/AlN/GaN stack structures will be reduced so that all 2DEG channels can be depleted and controlled under high reverse *V*_gs_ bias voltage conditions. In Figure 2d, the rocking curve X-ray signals were measured on the HEMT structures. The X-ray peaks were observed at 34.59° for the GaN layer, 35.02° for the AlGaN layer, and 36.09° for the bottom AlN buffer layer. The multiple peaks of the AlGaN channel layers were observed in the X-ray curves that indicated the high interface quality at the GaN/AlN/AlGaN channel-stacking structures.

**Figure 3:**
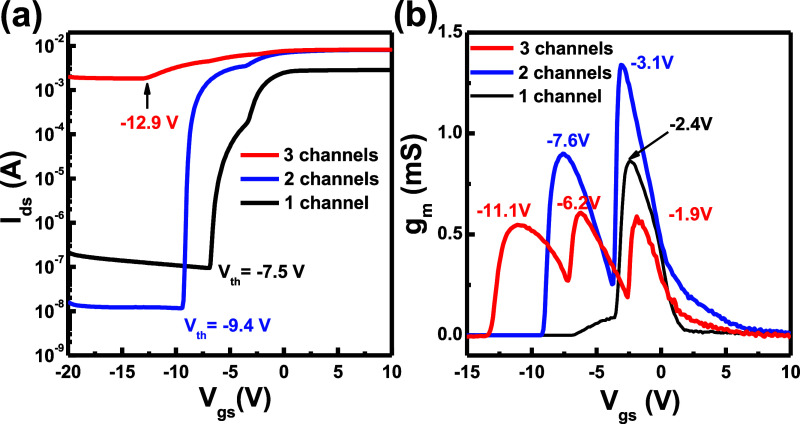
(a) *I*_ds_ currents as a function of *V*_gs_ were measured in all HEMT structures. (b) Transconductance spectra as a function of *V*_gs_ were measured in all HEMT structures at room temperature.

In Figure 3a, the *I*_ds_ currents, with log scale, as the function of gate voltage (*V*_gs_) were measured at room temperature. The *V*_th_ voltage of the two-channel structure was higher than the one-channel structure, so the deep 2DEG channel needs a high reverse gate bias voltage to deplete it. For the three-channel HEMT structure, the conductive channel was slightly reduced at −12.9 V with 1.85 mA so that the three channels cannot be closed in this epitaxial structure. A higher current at the on-state and lower current at the off-state were observed in the two-channel HEMT structure compared to one- and three-channel HEMT structures. The high leakage current in the three-channel structure was caused by the bottom third 2DEG channel not depleting under high *V*_gs_ bias voltage. By reducing the thickness of the GaN channel epitaxial layer, two GaN/AlN/AlGaN channel structures can be inserted in the conventional one-channel structure to improve the electron density in the 2DEG structure and enhance the conductive current operated in the active state. In Figure 3b, the transconductance spectra of the channel-stacking structures were measured by varying the gate voltages. The peak *V*_gs_ voltage and the transconductance values (*g*_m_) were operated at +20 V for *V*_ds_ by varying the *V*_gs_ bias voltage as shown in Figure 3b. In a one-channel structure, the peak *g*_m_ value was measured as 0.86 mS at −2.4 V (*V*_gs_) for the conventional HEMT device. The peak *g*_m_ values and voltage were measured as 1.34 mS (−3.1 V)/0.90 mS (−7.6 V) for the two-channel structure and 0.59 mS (−1.9 V)/0.61 mS (−6.2 V)/0.55 mS (−11.1 V) for the three-channel structure. This indicated that each channel in the multiple-channel structure was operated by increasing the reverse gate bias voltage. The peak *g*_m_ values of the two-channel structure were higher than those of the conventional one-channel structure. All peak *g*_m_ values of the three-channel structure were lower than those of the one- and two-channel structures due to the large leakage current at the reverse gate bias condition. The conductive properties of the channel-stacking structures were observed clearly in the *g*_m_ spectra. Higher *I*_ds_ current and lower pinch-off current were observed in the two-channel structure compared with the conventional one-channel structure at room temperature. The threshold voltage (*V*_th_) value is defined by the equation *I*_ds_ = *K*(*V*_gs_ – *V*_th_)^2^ operated in the active region. After the root of the *I*_ds_ current curve operated in the active region, the tangent interception at the *V*_gs_ axis is defined as the *V*_th_ value. The *V*_th_ values and off currents were measured as −7.5 V/9.64 × 10^–8^ A for one-channel and −9.4 V/1.19 × 10^–8^ A for two-channel HEMT structures. The *V*_th_ voltage of the two-channel structure was higher than that of the one-channel structure and the deep 2DEG channel needed a high reverse gate bias voltage to deplete it. For the three-channel HEMT structure, the conductive channel was slightly reduced at −12.9 V with 1.85 mA such that the three channels could not be closed in this epitaxial structure. A higher current at the on-state and lower current at the off-state were observed in the two-channel HEMT structure compared to one- and three-channel HEMT structures. By reducing the thickness of the GaN channel epitaxial layer, two GaN/AlN/AlGaN channel structures can be inserted in the conventional one-channel structure to improve the electron density in the 2DEG structure and enhance the conductive current operated in the active state. In Figure 3b, the transconductance spectra of the channel-stacking structures were measured by varying the gate voltages. In the one-channel structure, the peak *g*_m_ value was measured as 0.86 mS at −2.4 V (*V*_gs_) for the conventional HEMT device. The peak *g*_m_ values were measured at −3.1 V/–7.6 V for the two-channel structure^5,21^ and −1.9 V/–6.2 V/–11.1 V for the three-channel structure. This indicated that each channel in the multiple-channel structure was operated by increasing the reverse gate bias voltage. The peak *g*_m_ values of the two-channel structure were higher than those for the conventional one-channel structure. All peak *g*_m_ values of the three-channel structure were lower than the one- and two-channel structures due to the large leakage current at the reverse gate bias condition. The conductive properties of channel-stacking structures were observed clearly in the *g*_m_ spectra. Higher *I*_ds_ current and lower pinch-off current were observed in the two-channel structure compared with those on the conventional one-channel structure.

**Figure 4:**
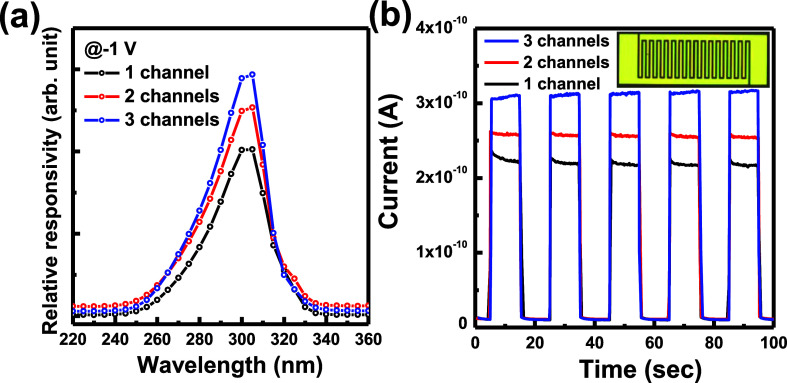
(a) Responsivities of all PD devices were measured by varying the wavelengths of the incident monochromic light. (b) Transient response photocurrents of the PD devices were measured by using the 300 nm monochromic light.

In Figure 4a, the relative responsivities of the AlGaN/AlN/GaN channel-stacking PDs were measured by varying the wavelengths of the incident monochromic light at a −1 V bias voltage. The peak wavelengths of the responsivity spectra were located at about 300 nm for all AlGaN/AlN/GaN channel-stacking PD structures. The illuminated light intensities were measured using a Newport power meter (1830C) for the power intensity as a function of the wavelength. The device’s size was 800 μm × 270 μm with a 9 μm width, 9 μm spacing, and 200 μm length in the finger-type electrode. The relative responsivities of the PD structures are shown in Figure 4a for comparing the device performance with different numbers of the 2DEG channels. The cutoff wavelength of the responsivity spectra was observed at 340 nm for the PD structures due to the band-edge absorption of the GaN channel layers in the AlGaN/AlN/GaN 2DEG channel structure. In the PD structure, the top GaN cap layer is a very thin layer above the AlGaN/AlN/GaN stack structures, and the top MSM metal layer depletes the carrier in the GaN cap layer. For the AlGaN and GaN layers in the PD structure, the light absorption wavelengths are shorter than the bandgap of the AlGaN and GaN layers due to the band-edge absorption properties of the epitaxial layer. The peak wavelength of the relative responsivity spectra will be shorter than the peak wavelength in the PL spectra. The experiment results showed that the AlGaN layers’ absorption efficiencies were higher than those of the GaN layer in the AlGaN/AlN/GaN stacking channel PD structures. Compared with the one-channel structure, the enhanced ratios of the peak responsivity were calculated as values of 1.25 times for the two-channel structure and **1.45 times** for the three-channel structure. For the transient response measurement in Figure 4b, the chopper time is 10 s open and 10 s close to control the incident 300 nm monochromic light. The on/off time was 10 s/10 s to analyze the current at a −1 V bias voltage. The photocurrent and the dark current were measured as 2.18 × 10^–10^/1.01 × 10^–11^ A, 2.57 × 10^–10^/1.11 × 10^–11^ A, and 3.13 × 10^–10^/1.03 × 10^–11^ A for the one-channel, two-channel, and three-channel PD structures, respectively. All of the channel-stacking PD structures had similar dark currents in the off states. Compared with the photocurrent of the one-channel structure under 300 nm incident monochromic light, the enhanced ratios of the photocurrent were calculated as **1.18 times** for the two-channel structure and **1.44 times** for the three-channel structure. The photocurrents were increased by adding a number of 2DEG channels, in which the illuminated light was absorbed by each channel in the AlGaN/AlN/GaN channel-stacking structure. However, the photocarrier generated at the deep channel was not totally transported to the top metal contact layers in MSM-PD structures.

## 4 Conclusions

The AlGaN/AlN/GaN channel-stack HEMT structures were demonstrated. The saturation *I*_ds_ currents were increased by adding a number of channels. The interference properties of AlGaN/AlN/GaN stack structures were observed in the X-ray curves, which indicated the high-quality interfaces of the conductive channels. The multiple channels’ conductive properties corresponded to the peaks of the *g*_m_ spectra in the HEMT structures at reversed gate bias conditions. The two-channel HEMT structures operated in the depletion mode were pinched-off by increasing the reversed *V*_gs_ voltages. The photocurrents were increased by adding a number of 2DEG channels such that the illuminated light was absorbed by each channel in the AlGaN/AlN/GaN channel-stacking structure. A higher current in the active state, a lower current at the cutoff state, a high *g*_m_ value, and a high transient response photocurrent were observed in the two-channel HEMT structure compared with the one- and three-channel HEMT structures.
